# Supplementary material for: MViT: A vision transformer with fractal path reordering and dynamic positional encoding
Source: PLoS One. 2026 Jan 16;21(1):e0340788. doi: 10.1371/journal.pone.0340788 (PMC12810909; doi:10.1371/journal.pone.0340788)
Supplement: Attachment — Submitted filename: Response to Reviewers.pdf [file pone.0340788.s001.pdf]

PONE-D-25-38700

MViT: A Vision Transformer with Fractal Path Reordering and Dynamic Positional Encoding

PLOS ONE

To the Academic Editor
Dear Dr. Anil Yaman,

We sincerely appreciate the time and effort devoted by you and the three reviewers to the evaluation of our submission entitled *"MViT: A Vision Transformer with Fractal Path Reordering and Dynamic Positional Encoding"* (Manuscript ID: PONE-D-25-38700).Your constructive observations and professional guidance have greatly contributed to improvements in the conceptual framework, experimental design, and overall clarity of this study. Each comment has been examined with great care, and corresponding revisions have been completed in accordance with its academic significance.

A clarification is provided regarding a previous misunderstanding related to the supporting information figures (S1–S10) mentioned in the editorial notice. Initially, the guidelines for supporting information were misinterpreted, and the Supporting Information section preset in the LaTeX template provided on the official ***PLOS ONE*** website was mistakenly understood as a place where the captions of figures already cited in the main text should be relocated. It is now fully understood that the primary purpose of supporting information is to provide additional materials that are relevant to the study but not directly cited in the main text. For figures that are cited in the main text, their captions must be embedded within the manuscript and placed immediately after the paragraph where they are first mentioned, in complete accordance with the explicit requirements of the journal.

In the revised version, the logical structure and methodological details of the proposed model have been refined. Additional experiments and data analyses have been included to confirm the effectiveness and cross-architecture compatibility of the MViT framework. The *Abstract*, *Introduction*, *Methods*, and *Results* sections have been thoroughly revised to achieve clear consistency among research motivation, methodological innovation, and empirical evidence. The entire text has undergone professional English editing to ensure clarity, precision, and full conformity with ***PLOS ONE*** publication requirements. All equations, figures, and visual elements have been carefully reviewed and optimized for accuracy and readability.

We are deeply grateful for the rigorous editorial process and the valuable guidance provided by the ***PLOS ONE*** editorial board. These comments have not only enhanced the quality of the paper but also provided meaningful directions for future investigations. The current submission includes the revised version of the paper and a

detailed point-by-point response document describing how every comment has been addressed.

In accordance with the data availability policy of the journal, the dataset and source code associated with this study have been deposited in the ***Figshare*** repository to facilitate public access and reproducibility. These materials are available at ***URL***: https://figshare.com/authors/Linjun_He/22524461. In addition, a funding statement has been added to the revised manuscript, clearly declaring that the funders had no role in the study design, data collection and analysis, decision to publish, or preparation of the manuscript. This clarification ensures the independence and academic integrity of the research.

We again extend our sincere thanks for the careful consideration of this work. We hope that the revisions adequately resolve all concerns and that the paper now meets the academic standards expected by ***PLOS ONE***.
With highest respect, we kindly invite you to review the revised version.

Yours sincerely,
Author Team
School of Design and Art, Shanghai Dianji University
November 15, 2025

Response to Reviewer 1

Dear Reviewer,

We sincerely appreciate your careful evaluation and thoughtful comments on our paper. Your observations reflect profound academic insight and strong technical relevance. They have precisely identified aspects that could be improved in model design and experimental validation, while providing valuable guidance for refining the logical organization of the study and extending the applicability of the proposed framework. Each suggestion has been carefully considered, and extensive revisions have been made to the structure, experimental coverage, and presentation of the paper.

In accordance with your guidance, the conceptual role of the proposed Moore Path and Periodic Positional Encoding modules has been clearly restated, with explicit definitions and theoretical clarification. Additional experiments have been included across different backbone architectures and tasks to confirm the generalization ability of the framework. The experimental scope now covers not only image classification but also object detection and segmentation. Comparative analyses with advanced positional encoding methods have been added, and all quantitative results are presented as averages with standard deviations obtained from multiple runs to ensure statistical reliability. The entire text has been carefully edited for grammatical precision, and all equations and figures have been verified for correctness and consistency.

We are sincerely grateful for your rigorous and constructive feedback, which encouraged us to review the scalability of our method and to strengthen the completeness of the experimental evaluation. Your comments have significantly improved both the theoretical depth and the practical contribution of this study.The following section provides detailed responses to each of the five comments raised in your review.

Comment	Response
1. The nature of the proposed method. The authors focused on improving patch ordering and encoding in the ViT architecture, without changing the downstream attention layers. Therefore, the proposed method can be used as an "add-on" module to a wide range of existing ViT architectures using traditional ordering and absolute encoding, rather than "replace" them. The authors should consider emphasizing that the proposed method can flexibly equip other ViTs with fancy attention block designs, which can bring additional predicting power. As a result, to present the experimental effectiveness, the authors should consider adding comparisons like "ViT vs. ViT+proposed Moore+PE", "Swin vs. Swin+proposed Moore+PE", etc. This could significantly extend the application.	We sincerely appreciate the valuable comments provided during peer review. In the revised version, the role of the Moore Patch Ordering and Periodic Positional Encoding (Moore+PPE) module has been clearly redefined. It is described as an embeddable input feature enhancement component that can be integrated into various Vision Transformer backbones without any modification to the attention-layer structure or the optimization configuration.<br><br>According to the review comments, Section 4.7 has been expanded to include comparative experiments using ViT-B/16 and Swin-T as representative backbones. The results compare the baseline models with those incorporating the Moore–PPE module, and the complete findings are summarized in Table 8. The experiments cover classification tasks on the ImageNet-21k and CIFAR-100 datasets and further assess generalization performance on downstream tasks, including object detection and instance segmentation, using the COCO 2017 dataset.<br><br>With only minor increases of 0.05 M to 0.22 M parameters and 0.06 G to 0.15 G FLOPs, the Moore+PPE module achieves stable and consistent performance improvements across different backbones and tasks. Accordingly, the Abstract, Method Overview, and Contributions sections have been revised to explicitly define it as an embeddable input feature enhancement component and to emphasize its structural compatibility and generalization capability across different tasks.
2. Computer vision tasks. Related to (1), as general ViTs can be combined with the proposed method, the enhanced models can still be used as the backbone of different types of CV tasks. Nevertheless, the authors focused on image classification tasks in this	We sincerely appreciate the valuable suggestion regarding task coverage. In the revised version, the experiments have been extended from classification and reconstruction to downstream object detection and instance segmentation. In Section 4.7, experiments were conducted on the COCO 2017 dataset using Swin-T as the backbone to systematically evaluate the adaptability of the proposed Moore+PPE module for detection and segmentation tasks. The results show that $AP_{(bbox)}$ for object detection increased from 43.6% to 44.1%, while $AP_{(mask)}$ for instance segmentation improved from 39.8% to 40.3%. The corresponding increases in parameters and computation were negligible, with the parameter count rising from 47.8 M to 48.02 M and

manuscript. While Section 4.3 is related to image reconstruction, the data set and settings in Table 3 are not clear. The authors should consider adding data sets and experiments for more common CV tasks such as object detection and instance segmentation (e.g., on COCO data set) to show the general advantages of the proposed module.	FLOPs increasing from 264.1 G to 264.25 G.<br><br>For the image classification experiments, additional comparative studies between the baseline backbones and the backbones integrated with the Moore+PPE module were included in accordance with the review comments. The results indicate that ViT-B/16 improved in Top-1 accuracy from 83.77% to 84.08% on ImageNet-21k and from 90.42% to 90.86% on CIFAR-100, while Swin-T improved from 84.02% to 84.32% on ImageNet-21k and from 89.83% to 90.21% on CIFAR-100 (see Section 4.7 and Table 8 for details). These findings collectively demonstrate that, without altering the backbone architecture or optimization settings, the Moore+PPE module provides stable and consistent performance improvements across classification, detection, and segmentation tasks, demonstrating strong structural compatibility and generalization capability.<br><br>To address the concern regarding unclear experimental settings, Section 4.1 has been supplemented and standardized to include detailed information about dataset types and partitioning. Specifically, Table 1 specifies the COCO 2017 configuration, including 80 categories and approximately 123,000 images, with 118,287 for training and 5,000 for testing. All experiments were implemented using PyTorch on an NVIDIA A100 platform. The training and validation data partitions were kept fixed, and each dataset was run five times using fixed random seeds {42, 123, 3407, 2023, 5566}. The reported results represent the mean and standard deviation across five runs, with standard deviations ranging from 0.12% to 0.37%, ensuring both stability and reproducibility.
3. Baseline models. In Table 2, all the 3 baseline models use traditional absolute positional encoding which is known to be weak in cases like image rotation. As the periodic encoding usage is a novelty in this paper, the author should consider adding a baseline model using stronger encoding methods as well (e.g., relative/rotary encoding [1]).<br>In addition, while the Swin-T selection for Swin transformer is clear, what is the specific size of vanilla ViT (e.g., L/16) used in Table 2 and 3? If the parameter numbers are not comparable	We sincerely appreciate the valuable comments regarding the baseline models. In response, the revised manuscript provides a comprehensive clarification and update to the descriptions of baseline model scale, positional encoding types, and the compatibility of the proposed Periodic Positional Encoding (PPE). In the updated Section 4.1 Datasets and Experimental Settings, we explicitly stated:<br>***The baseline models presented in Tables 2 and 3 adopted their default publicly available positional encoding schemes. ViT-B/16 used absolute positional encoding, while Swin-T incorporated relative position bias, representing a stronger spatial modeling baseline. The parameter sizes of ViT-B/16 and Swin-T were approximately 86M and 28M, respectively, which fall within the typical range for their corresponding architectures, ensuring comparability of results across different structures.***<br>This clarification identifies the ViT variant used in this work as ViT-B/16 and supplements details on model scale and comparability, thereby addressing the

between different models, the results won't make sense.<br><br>[1] Heo, Byeongho, et al. "Rotary Position Embedding for Vision Transformer." European Conference on Computer Vision. 2024.	comment requesting specification of the ViT size and parameter consistency.<br><br>Within the same section, three mainstream positional encoding mechanisms were further explained, highlighting their distinctions and complementarity. The revised manuscript now includes the following statement:<br>***The proposed periodic positional encoding and relative position bias are complementary in mechanism and can be jointly applied within the same framework. In subsequent experiments, PPE maintained stable performance improvement under the relative bias structure of Swin-T, as shown in Table 8, demonstrating compatibility with stronger positional encoding forms***.<br>This revision clarifies that PPE remains effective even when integrated into stronger encoding baselines, thus addressing the suggestion to evaluate it with more advanced encoding methods.<br><br>In addition, the Related Work section has been expanded to describe the role and mechanism of the PPE module, illustrating its scalability and structural independence. The revised text reads:<br>***The approach proposed in this work combines a Moore traversal path with a periodic positional encoding module(PPE). The proposed module encodes tokens along the Moore path through a periodic parameter T and controlled phase modulation, which enforces coherent positional progression. This design acts as a lightweight component at the input stage and can be inserted into different ViT backbones without any change to the attention layers or the task heads. In this way, it improves robustness to local structural patterns and rotated content.***<br>This definition highlights that PPE can be integrated into multiple backbones without altering attention structures, reinforcing reusability and practical generality. To verify the effectiveness of PPE across different backbone architectures, Section 4.7 Cross-Backbone Compatibility and Lightweight Evaluation introduces new experiments. These experiments confirm stable performance gains and extremely low computational cost for PPE within the Swin-T architecture that incorporates relative positional bias, demonstrating robustness and scalability under stronger encoding frameworks.<br>In summary, through additions to methodology, experimental settings, and result analysis, the revised manuscript clarifies the ViT backbone scale and parameter comparability, extends the comparison framework for positional encodings, and validates the compatibility and effectiveness of PPE under strong baseline conditions.

4. Error bar. In the result tables, most of the accuracy enhancement brought by the proposed method are within 1%. The authors should consider reporting the standard deviation of the performance metrics, to show the stability and reproducibility of the performance.	We sincerely appreciate the valuable comments concerning the statistical reliability of the experimental results. In the revised manuscript, we added explicit details on the statistical procedures for multiple independent runs and reported all quantitative results in the tables as mean values with standard deviations. In the updated Section 4.1 Datasets and Experimental Settings, the following statement has been included:<br>***To ensure stability and reproducibility, each dataset was trained and evaluated independently five times with fixed random seeds {42, 123, 3407, 2023, 5566}. The reported results in all tables represent the mean values of the five independent runs, with standard deviation ranging from ±0.12% to ±0.37%. This configuration ensured stable and reproducible performance under different initialization conditions.***<br><br>This revision clarifies that all reported results are based on the average across five independent experiments, and that the standard deviation range reflects variation arising from different random initializations. Compared with the original version, the revised manuscript unifies the random-seed settings and the procedures for repeated experiments across all datasets, thereby ensuring statistical consistency and reproducibility of the comparative results.<br><br>Furthermore, the results presented in Tables 2 through 8 were obtained under an identical experimental configuration. The observed performance differences fall within the fluctuation range of the five independent runs, demonstrating the stability and reproducibility of the outcomes. These results confirm that the proposed MViT model maintains consistent generalization performance across multiple tasks. Through these additions, the revised manuscript systematically demonstrates the experimental stability and performance reliability of the proposed model.
5. Writing. Please check the writing and fix the typos (e.g., excel -> excels in the abstract).	We sincerely appreciate the reviewer for the detailed comments on language quality and spelling accuracy. The entire manuscript has undergone a comprehensive language review and revision, with particular attention to grammatical consistency, tense uniformity, and the standardization of technical terminology in the Abstract, Introduction, and Experimental sections.<br><br>Beyond this correction, we carefully reviewed the entire manuscript for agreement between subject and verb, tense consistency, and proper

	pluralization of nouns, and we made the necessary revisions. The terminology across the paper has also been standardized to ensure academic precision and consistency.<br><br>The revised manuscript has been thoroughly checked using professional language editing software and manual proofreading to ensure that the grammar, spelling, and formatting meet international academic publishing standards. We thank the reviewer for the constructive suggestions, which have improved the linguistic rigor and clarity of the manuscript.

Response to Reviewer 2

Dear Reviewer,

We sincerely appreciate your careful review and detailed feedback on our manuscript. Your comments are precise and insightful, and they identify valuable directions for improvement in the structure, clarity of expression, and experimental presentation of the work. They provide important guidance for enhancing the academic depth and overall quality of the study. We have given full consideration to each suggestion and, based on a thorough understanding of the key points, have made systematic revisions to the title, the introduction, the research motivation, the mathematical formulations, the experimental analysis, and the language and the quality of figures.

During the revision process, we reexamined the overall logic of the manuscript and refined the connection between the introduction and the methodology in order to present the research questions and the innovations in a more focused and coherent manner. Following your suggestions, we expanded the introduction by incorporating the latest advances in visual Transformer research across multiple domains, including representative studies in medical imaging and smart agriculture. This enhancement increased both the academic depth and the practical relevance of the study background. We also rewrote the sections on research motivation and the summary of literature gaps to ensure more consistent logic and a clearer definition of the research problem. Regarding mathematical and structural formulations, we carefully verified the symbols and the logical relationships in Equations (3) to (8), added complete variable definitions and parameter constraints, and ensured precision and consistency throughout the mathematical descriptions.

In terms of results and presentation, we extended the discussion section with additional analyses of the mechanisms that explain the improvement in model performance and its generalization across different tasks. We clarified the role of the periodic modulation structure in modeling symmetry and maintaining structural continuity. Furthermore, we refined the language of the manuscript, corrected grammatical and typographical errors, and replaced all figures and tables with high resolution versions to meet academic publication standards for readability and visual quality.

We sincerely thank you for your rigorous and constructive comments, which helped us refine the logical structure and the theoretical exposition of the manuscript. Your feedback significantly improved the scientific robustness and the general applicability of the research.The following section provides a detailed point by point response to each of the eight comments.

Comment	Response
1. The title of this manuscript has not effectively captured the interest of a broader audience outside its niche area, which will limit its impact and reach, please revised	We sincerely thank the reviewer for the valuable comments. A clear and communicative title is essential for improving both the influence and the readability of a paper. The original title, ***MViT: Dynamic Positional Encoding Vision Transformer Guided by Recursive Moore Curves***, contained specialized terminology such as recursive Moore curves, which may not be easily understood by readers outside the Transformer community and could therefore limit the cross disciplinary accessibility of the work.<br><br>In response to this comment, we carefully analyzed the composition and communicative impact of the title. To enhance clarity and readability while maintaining technical accuracy, we decided to simplify and optimize the wording so that it is more intuitive and accessible to readers from various research domains. The revised title, ***MViT: A Vision Transformer with Fractal Path Reordering and Dynamic Positional Encoding***, reflects these improvements.The new title offers several advantages. The term fractal path reordering preserves the core innovation while using terminology that is more widely recognized and easier to interpret in computer vision and pattern recognition fields. The term dynamic positional encoding accurately represents the proposed mechanism of periodic and adaptive positional modulation, which allows readers to understand the key methodological feature of the approach more directly.
2. The introduction can be enriched by briefly describing the state-of-the-art in the title of this study and providing more recently related references to support the foundation of this study and provide a better context for the current research. I will recommend citing recently published related papers;<br>a. "Semantic Segmentation of the Lung to Examine the Effect of COVID-19 Using UNET Model," in Applied Machine Learningand Data Analytics. AMLDA 2022.	We sincerely thank the reviewer for the valuable comments. In response to the suggestion that the introduction should include the latest research progress and related references, we have systematically expanded the research background in the revised manuscript. In particular, we added a review and citations of recent studies on cross domain applications of visual Transformers in Section 1 of the introduction.<br><br>We carefully reviewed and adopted two representative references recommended by the reviewer. These studies represent the latest advances in semantic segmentation in medical imaging and behavior recognition in intelligent agriculture, and they demonstrate the generalization potential and adaptability of this architecture across different task scenarios. In the revised introduction, we added the following paragraph:<br><br>***These methods have enhanced the performance of visual Transformers in***

Communications in Computer and Information Science, 2023.<br>b. "Vision transformers for automated detection of pig interactions in groups," Smart Agricultural Technology, vol. 10, p. 100774, 2025.	***maintaining spatial structures and modeling geometric relationships, demonstrating consistent structural expression across different tasks. For example, in medical imaging research, the U-shaped convolutional neural network (U-Net) model has been employed for lung signal slicing, enabling precise modeling of Coronavirus Disease 2019 (COVID-19) lesions. In smart agriculture, visual Transformers have been used for the automated detection of pig group interaction behaviors., indicating that such architectures maintain stable feature representations and robust generalization in complex visual environments.***<br><br>This revision not only supplements the latest research progress of visual Transformers in cross domain applications but also makes the technical background of this study more complete, strengthening its relevance for structural modeling and geometric generalization. We sincerely appreciate the constructive suggestion from the reviewer, which significantly improved the academic depth and literature foundation of the introduction section.
3. The problem that the author is trying to address in this paper is not clear yet, (clearer motivation for the study is required)	We sincerely thank the reviewer for the valuable comments on the explanation of the research motivation. We fully agree that the statement of the research problem in the earlier version was not sufficiently focused and did not clearly present the underlying motivation and theoretical foundation. Following the reviewer recommendation, we systematically adjusted the logical connections among the abstract, the introduction, and the experimental sections in the revised manuscript, ensuring that the research motivation, the theoretical assumptions, and the experimental validation form a coherent framework.In the abstract, we added the concluding sentence:<br>***Ablation and rotational perturbation experiments further confirm its robustness to rotation and localized focus variations. Moreover, MViT exhibits strong structural compatibility, maintaining stable performance across different Transformer backbones and diverse visual tasks.***<br><br>This revision enriches the discussion of model robustness and generality, and directly links the research motivation with the experimental validation, forming a complete logical progression from problem formulation to performance demonstration. In the introduction, we strengthened the logical expression of the research motivation and the

problem definition, clearly identifying three main limitations of existing methods and proposing corresponding improvements:

Although existing studies have made progress in local structure modeling and positional encoding, three major limitations remain. First, fixed sequential traversal paths weaken spatial adjacency between patches and reduce local continuity. Second, most methods rely on input resolutions that are powers of two, which makes it difficult to maintain consistent boundary alignment for irregular image sizes. Third, conventional absolute or relative positional encodings lack periodic modulation, leading to phase mismatch and index mapping mismatch under rotations, symmetries, or scale changes, thereby limiting structural representation and geometric generalization in complex visual tasks. To address these issues, this study proposes MViT, a recursive framework based on fractal mapping that jointly optimizes dynamic curve mapping and positional encoding to enhance local structure modeling and spatial awareness.

This addition establishes a clear correspondence between the research motivation and the proposed framework design, and it naturally introduces the three core modules of MViT, namely Moore path construction, periodic positional encoding, and a boundary compensation mechanism.

Moreover, Sections 4.2 to 4.4 of the experimental part further verify the proposed motivation through evaluations on multiple datasets and through ablation studies. For instance, in the rotation perturbation experiment on CIFAR 100 (Table 3), the accuracy reduction of MViT was 12.6 percent smaller than that of ViT, which confirms its stability in rotational structure modeling. In the image reconstruction task (Table 4), the PSNR increased to 32.45 dB and the SSIM reached 0.921, which verifies that the path and boundary mechanisms improved structural continuity. The ablation results in Tables 5 and 6 quantitatively demonstrate the independent contribution of the periodic positional encoding to structural focus and semantic alignment.

Thus, the revised manuscript establishes a complete logical chain among the research motivation, the problem definition, and the experimental validation, presenting the research question, the technical innovation, and the empirical evidence more clearly. We sincerely appreciate the valuable

	comments from the reviewer, which significantly improved the scientific logic and the depth of argumentation in this study.
4. In 4-5- lines, authors should summarize the literature gaps identified before starting the methodology (Kindly relate your work in the context of existing work, what are the shortcomings of existing studies?)	We sincerely thank the reviewer for the valuable comments on the explanation of the research motivation. In the revised manuscript, we added a new paragraph in the introduction, placed before the methodology section, to summarize frontier studies and the remaining research gaps, and we clarified how the experimental design systematically verifies these limitations.<br><br>First, we introduced representative references from different research domains to highlight both the application value and the current limitations of visual Transformer frameworks in complex scenarios.<br>***These methods have enhanced the performance of visual Transformers in maintaining spatial structures and modeling geometric relationships, demonstrating consistent structural expression across different tasks. For example, in medical imaging research, the U-shaped convolutional neural network (U-Net) model has been employed for lung signal slicing, enabling precise modeling of Coronavirus Disease 2019 (COVID-19) lesions. In smart agriculture, visual Transformers have been used for the automated detection of pig group interaction behaviors., indicating that such architectures maintain stable feature representations and robust generalization in complex visual environments .***<br><br>Second, following this literature summary, we explicitly outlined the research gaps as follows:<br>***Although existing studies have made progress in local structure modeling and positional encoding, three major limitations remain. First, fixed sequential traversal paths weaken spatial adjacency between patches and reduce local continuity. Second, most methods rely on input resolutions that are powers of two, which makes it difficult to maintain consistent boundary alignment for irregular image sizes. Third, conventional absolute or relative positional encodings lack periodic modulation, leading to phase mismatch and index mapping mismatch under rotations, symmetries, or scale changes, thereby limiting structural representation and geometric generalization in complex visual tasks.***

	This revision systematically summarizes the limitations of previous studies and provides a logical foundation for the innovative design of MViT. In addition, we extended Sections 4.2 to 4.9 in the experimental part to demonstrate that these research gaps have been addressed through empirical evidence. For instance, the ablation experiments confirmed that periodic positional encoding effectively mitigates the rotation sensitivity of conventional absolute encodings, while the cross backbone evaluation in Table 8 verified model transferability in detection and segmentation tasks on the COCO dataset, addressing the lack of cross task generalization analysis in the existing literature.
5. Author should re-check the correctness of equations, especially from equations 3 to 8	We sincerely thank the reviewer for pointing out potential issues in the formula section. We carefully reexamined the symbol definitions, structural logic, and parameter dependencies for Equations (3) to (8) and found that the constraints among certain variables and the description of the periodic modulation mechanism in the earlier version were not sufficiently rigorous, which could lead to ambiguity. In response to this comment, we conducted a systematic revision of Equations (3), (4), (6), (7), and (8).<br><br>Following this revision, the definitions and logical relations of Equations (3), (4), (6), (7), and (8) have been corrected and unified. Specifically, Equation (3) now states an integer multiple constraint between the period parameter and the path length, so that periodic modulation strictly corresponds to path traversal. Equation (4) refines the definitions of the embedding dimension and the phase offset, ensuring that frequency control and phase adjustment within positional embeddings are self consistent. Equation (6) adds direction alignment and offset mechanisms to preserve index continuity when concatenating the main path with the compensation path. Equation (7) sets the end point of the main path as the phase reference, achieving phase consistency between paths. Equation (8) introduces explicit terms for frequency fine tuning and phase calibration, ensuring a smooth transition of periodic embeddings between the compensation segment and the main path. These changes improve the logical integrity and explainability of the mathematical formulation and make the presentation of periodic positional encoding and boundary compensation more rigorous. The revised content has been incorporated into Sections 3.3 and 3.4 of the paper.

6. More discussion of the results are required	We sincerely thank the reviewer for the valuable comments regarding the depth of the result discussion. In the revised manuscript, we expanded Section 4.9 by adding a systematic analysis of the mechanisms behind the performance improvement of the model and the observed differences in the results.<br><br>First, in terms of structural mechanisms, we added an explanation of the periodic positional encoding module. This module enhances the attention response to symmetric structures and fine grained targets by integrating shallow convolutional features with periodic rhythmic modulation.<br><br>Second, at the level of result analysis, we supplemented the discussion of performance differences across datasets. The revised version indicates that MViT achieved higher Top 1 accuracy than ViT-B/16 and Swin-T by 0.31 percent and 0.52 percent on the ImageNet-21k and CIFAR-100 datasets, respectively, and reached 90.75 percent on the Stanford Cars dataset. The PSNR and SSIM values increased by 8.8 percent and 4.4 percent compared with GFPE-ViT, which confirms the contribution of the boundary compensation mechanism to spatial continuity modeling.<br><br>In addition, the new parameter sensitivity analysis shows that the model achieves its best performance when the fractal order t and the period parameter T satisfy an integer multiple relationship. This result reflects the coordinated effect between path topology and periodic modulation. We also added a discussion of the compatibility of the Moore+PPE module across different backbones. The module produced consistent gains on both the ViT B/16 and Swin-T architectures with minimal computational cost. Finally, we added a statement on the limitations of the method and possible future improvements. In particular, for high resolution scenarios, model efficiency may be further improved by incorporating dynamic path generation and conditional attention mechanisms.
7. Services of language experts are required (be mindful of tenses, typo errors)	We sincerely thank the reviewer for the attention to the language quality of the manuscript. In response to this comment, we carefully revised the entire manuscript and conducted a comprehensive review of tense consistency, spelling accuracy, grammatical correctness, and precision of wording. The revised version uses clearer and more standardized language and is consistent with academic writing conventions.

8. Image quality needs to be improved (High resolution image suggested)	We sincerely thank the reviewer for pointing out the issue of insufficient image resolution in the manuscript. After receiving this comment, we redrew and reformatted all figures and visual results in the manuscript, and we used high resolution images of at least 300 dpi to ensure clear detail and standardized annotations when enlarged. In addition, we optimized the color contrast and font size so that the figures maintain good legibility in both black and white printing and electronic viewing environments. All revised high resolution figures have been incorporated into the manuscript. We sincerely appreciate this valuable suggestion, which has significantly improved the visual presentation and layout quality of the manuscript.

Response to Reviewer 3

Dear Reviewer,

We sincerely appreciate your careful review and constructive suggestions. Your comments, which address convergence efficiency, computational cost, robustness under rotational and symmetric conditions, visualization of positional encoding, selection of baseline models, ablation analysis, statistical reproducibility, and presentation consistency, have provided valuable guidance for improving both the experimental design and the overall presentation of this study. Each suggestion has been examined in detail, and the manuscript has been revised accordingly with full consideration of the academic intent of your remarks.

In the revised version, a quantitative analysis of convergence rate and memory consumption has been included. The results indicate that the proposed MViT achieves approximately 30 percent faster convergence than ViT-B/16 when trained on relatively small datasets. Section 4.5 now provides a detailed description of the assessment of training efficiency. In response to the request for verification of performance under rotational transformations, Section 4.2 has been supplemented with a quantitative comparison based on rotation perturbation experiments on the CIFAR-100 dataset. These results confirm consistent performance and stable generalization under rotated and symmetric image configurations. In addition, visual analyses of positional encoding have been incorporated to illustrate the similarity distribution of the periodic positional encoding within a two dimensional structure. The contribution of each functional component has also been reorganized and presented in tables and figures, improving the clarity of the structural analysis.

Section 4.1 now provides a unified description of the statistical procedure applied in all experiments. Each configuration was executed five times on an NVIDIA A100 platform using a fixed random seed, and the results are reported as the mean value together with the standard deviation. This presentation is intended to ensure reproducibility and reliability. Details of data reporting have also been reviewed and corrected, including the total image count in the Places365 dataset, missing results for Swin-T, and inconsistent table annotations. These corrections ensure the accuracy and coherence of all reported data. The entire manuscript has been revised with respect to language and formatting, including tense, terminology, and spelling, in order to maintain precision and academic formality.

We sincerely appreciate your insightful and rigorous evaluation. Your comments have led to a comprehensive reassessment of efficiency, robustness, and completeness within the experimental framework, and have resulted in substantial improvement in both the theoretical foundation and the empirical validation of this study. The following sections provide detailed explanations of the revisions made in response to each of your comments.

Comment	Response
1.On page 2 you claim that you have 30% faster convergence speed (coming from figure 7 I believe).<br>Does your method takes more time to train compared to the others?<br>Does it takes more GPU memory then the others (model parameters)?	We sincerely thank the reviewer for the careful and insightful comments regarding the issues of training efficiency and computational overhead. We fully recognize the importance of this point and have thoroughly verified and supplemented the basis of the convergence acceleration conclusion, the experimental conditions, and the parameter scale in the revised manuscript.<br><br>In the updated Section 4.5, the comparability of the controlled experiments has been clarified. As revised,<br><br>***Figure 7 compares the training loss variations of MViT, ViT-B/16, Swin-T, and GFPE-ViT on ImageNet-21k and CIFAR-100 datasets. All models were trained for 100 epochs using the same batch size, learning rate, and GPU utilization conditions. Compared to other models, MViT converged faster within the first 40 epochs, with a steeper overall loss curve and lower final error***.<br><br>The reported approximately 30 percent acceleration refers to the reduction in the number of iterations required to reach the same loss level under identical training settings, rather than an increase in computational resources.<br><br>In response to the issue of memory usage and parameter scale, Section 4.7 now includes detailed quantitative data:<br><br>***In the image classification task, the Top-1 accuracy of ViT-B/16 improved from 83.77% to 84.08% on ImageNet-21k, and from 90.42% to 90.86% on CIFAR-100. The Top-1 accuracy of the Swin-T backbone on the same tasks increased from 84.02% to 84.32%, and from 89.83% to 90.21%, respectively. The parameter count of both experiments increased by only approximately 0.05M to 0.09M, and the FLOPs increased by about 0.06G to 0.08G, with a minimal increase in computational cost.***<br><br>***To test the adaptability of the module in downstream visual tasks, object detection and instance segmentation evaluations were conducted on the COCO 2017 dataset using Swin-T as the backbone. The results show that the average precision (AP bbox) for detection tasks increased from 43.6% to 44.1%, and the average precision (AP***

	mask) for segmentation tasks increased from 39.8% to 40.3%. Correspondingly, the parameter count increased by approximately 0.18M to 0.22M, and the FLOPs increased by about 0.12G to 0.15G.<br><br>Furthermore, Section 4.1 has been revised to include details about the experimental platform and repeated validation. All experiments were conducted on an NVIDIA A100 platform, and the performance metrics reported in the tables represent the average results of five independent runs. The standard deviation ranged from 0.12 percent to 0.37 percent, ensuring stability and reproducibility of the experimental results.<br><br>This valuable comment has encouraged a more rigorous verification of the comparability of experiments and a clearer interpretation of the results, which have now been fully addressed in the revised manuscript.
2. On page 2 you say: " Visualization analysis showed the model's identification error for rotational targets decreased by 12.6%. To me this is not explained / supported in the paper. Could you follow up on this?	We thank the reviewer for pointing out this issue. After carefully reexamining the original manuscript, we found that the previous statement that the recognition error on rotated targets was reduced by 12.6 percent was not precise. This conclusion did not originate from visual analysis but was derived from the quantitative results of the rotation perturbation experiment. In the revised manuscript, this statement has been corrected, and the supporting evidence has been moved to Section 4.2. The revised text is as follows:<br><br>***Geometric stability of the model was validated using the CIFAR-100 rotated perturbation subset. During testing, image samples were randomly rotated within a range of 0 to 45 degrees to evaluate the robustness of the model to external geometric transformations. The reported accuracy represents the overall performance of the perturbed sample set, reflecting the adaptability of the model to rotational perturbations across multiple angles. Table 3 shows the accuracy changes of each model before and after perturbation. Compared to ViT-B/16, MViT exhibited a 12.6% reduction in accuracy drop, demonstrating that periodic positional encoding can maintain phase continuity under rotation, alleviating absolute positional index mismatches, and thus improving the stability and generalization ability of the model for rotational and symmetric structures.***<br><br>The experimental data in Table 3 show that the accuracy of ViT-B/16 decreases from 93.30 percent to 86.79 percent, while the accuracy of

<table>
<tr><td></td><td>MViT decreases from 93.82 percent to 88.17 percent. The reduction in performance is therefore smaller by 12.6 percent for MViT. This value was obtained directly from experimental measurements rather than from qualitative visualization.

Accordingly, the original wording referring to visual analysis has been removed in the revised manuscript. The conclusion is now presented in the experimental section using quantitative results together with an explanation of the underlying mechanism, to ensure consistency between the argument and the supporting evidence. We appreciate this comment, which has helped improve the precision and clarity of the manuscript.</td></tr>
<tr><td>3. As you claim that your method is better on rotational , symmetrical objects, would it be an idea that you run an experiment for this? Could you find a dataset with mostly rotational , symmetrical objects, and compare your classification score compared to others?</td><td>We sincerely thank the reviewer for the valuable suggestion. We fully recognize the importance of dedicated validation on rotated and symmetric targets, and corresponding explanations and extended experimental results have been included in the revised manuscript.

In Section 4.2, Table 2 presents a systematic comparison between MViT and several representative backbone models across datasets with different levels of structural complexity. The results show that MViT achieves consistent performance improvements on ImageNet-21k, CIFAR-100, Places365, iNaturalist2021, and Stanford Cars. In particular, on the Stanford Cars dataset, which is structure sensitive and fine grained, MViT reaches an accuracy of 90.75 percent, which is significantly higher than that of the baseline models. These findings indicate that the proposed Moore curve based patch sequencing and periodic positional encoding improve the ability of the network to represent complex geometric structures and local symmetric features.

Furthermore, in the same section, we introduced a rotation perturbed subset of CIFAR-100, with rotation angles not exceeding 45 degrees, as a geometric robustness test set to indirectly evaluate the adaptability of the model to rotated objects. The experimental results show that the reduction in accuracy of MViT is 12.6 percent smaller than that of ViT-B/16, indicating that MViT maintains higher structural stability and feature continuity under rotation. This result provides experimental evidence supporting the advantages of the model when dealing with rotated and symmetric structures. We again thank the reviewer for this</td></tr>
</table>

	helpful suggestion.
4. The original VIT paper (https://arxiv.org/abs/2010.11929) has a figure (figure 7 in the middle) on their learned positional embeddings, by this they show what the embeddings have learned, and that they correspond to the image patch. Could be a nice additional to your paper as well.	We thank the reviewer for this valuable suggestion. In response to the comment, we have added a visualization of positional embedding similarity for the MViT model as Figure 10 in the revised manuscript to examine its spatial encoding properties.<br><br>This visualization is based on cosine similarity and shows the positional embedding distribution learned by MViT under a 7×7 patch division. The presentation follows the same format as the middle panel of Figure 7 in the original ViT paper (Dosovitskiy et al., 2020). The results show that MViT produces a smooth and symmetric spatial distribution along both the row and column directions, with high continuity of similarity between adjacent patches. This pattern indicates that the periodic modulation mechanism captures positional correspondence within a two dimensional spatial structure.
5. You compare yourself to ViT[1] (2022), Swin-T[2](2021), GFPE-ViT[5] (2024). Aren’t there no other recent models as some of these are some years old.	We sincerely thank the reviewer for the valuable comments. In the revised manuscript, we have added a detailed explanation of the rationale for baseline selection and the scope of model coverage, in order to ensure the representativeness and scientific validity of the comparisons. The revision clarifies the selection criteria in Section 4.1 and demonstrates continued improvement over stronger positional encoding baselines in Section 4.2.In Section 4.1, the revised text is as follows:<br><br>***For consistent and reproducible comparison, three representative baseline models were selected, namely ViT-B/16 , Swin-T , and GFPE-ViT. These models correspond to three main positional modeling mechanisms: absolute positional encoding, relative position bias, and positional encoding based on fractal geometry. All three models provide public implementations, well defined architectures, and documented evaluation procedures, which supports reproducibility. Several recently introduced lightweight or task specific models, such as Efficient-ViT and HybridFormer, focus mainly on efficiency optimization or adaptation to specific domains. These models do not yet provide a unified classification benchmark across datasets, and therefore they were not included in the main comparison in this study.***

	This addition states that the compared models were selected to cover the three main categories of positional encoding mechanisms while ensuring reproducibility and consistency across different tasks.<br><br>Furthermore, Section 4.2 expands on the rationale and representativeness of these comparisons. The revised text is as follows:<br><br>***Swin-T employs a relative position bias mechanism, which encodes the relative positional information in the x and y directions during the attention computation to capture local spatial relationships, thus providing stronger geometric structure modeling capabilities. This mechanism maintains feature distribution stability under translation and slight rotational perturbations, serving as a stronger reference baseline compared to absolute positional encoding. Based on this structure, MViT continues to exhibit consistent performance improvement, suggesting that periodic positional encoding and relative position bias are complementary in spatial representation and can maintain stable and effective performance within different positional modeling frameworks.***<br><br>This revision clarifies that the proposed Moore+PPE module is effective not only on earlier models such as ViT but also on the stronger Swin-T architecture, where it still provides consistent gains. These results support the compatibility and generalization ability of the proposed method across mainstream transformer architectures.
6. As you have 3 methods, it would be nice to see the results of your method in isolation. I believe you try to show this in table 4 and 5, but the numbers are exactly the same as in table 2 where you have put all your methods. Can you clarify?	We sincerely thank the reviewer for the valuable comments. In the revised manuscript, additional explanations have been added in Section 4.4 to clarify the functional roles and experimental correspondence of Tables 2, 5, and 6. The revised text is as follows:<br><br>***This section evaluates the independent contributions of each module in structural modeling through two sets of ablation experiments, focusing on the performance contributions of the path construction strategy and the fusion mechanism. Table 2 reports the overall performance of the complete MViT model across multiple datasets for comparison with the baseline networks. Tables 5 and 6 analyze the independent contributions by removing or replacing individual modules under the same training configuration. For clarity and representativeness, these tables only present results for the***

	ImageNet-21k and CIFAR-100 datasets, corresponding to large and medium to small scale classification tasks, respectively. Other datasets exhibit a consistent relative improvement trend under the same experimental settings, with differences controlled within the ±0.3% range, demonstrating stable performance across various data conditions.<br><br>This paragraph clarifies three key points.First, Table 2 presents the overall performance of the complete model for comparison with the backbone networks, while Tables 5 and 6 correspond to independent ablation experiments that analyze the effects of the path construction strategy and the positional fusion mechanism.Second, all three tables are based on the same datasets and training configurations. Therefore, the results of the complete MViT model remain identical across these tables and provide a consistent reference for comparison.Finally, the revised manuscript states that only the results on ImageNet-21k and CIFAR-100 are reported in detail to represent datasets of different scales, while the remaining datasets follow the same trend under identical experimental settings.
7. For all tables, it is unclear to me if your experiments have been run once or multiple runs (with seeds). I do not see any st. Dev scores as well. I cannot see if you are better compared to the others due to a lucky run now.	We sincerely thank the reviewer for the attention to experimental reproducibility and statistical reliability. In the revised manuscript, additional details have been included in Section 4.1 to describe the number of runs, the random seeds, and the statistical reporting procedure, in order to ensure both reproducibility and statistical validity of the results. All experiments were conducted using the PyTorch framework on an NVIDIA A100 platform. The training and validation data splits were kept fixed, and each dataset was run independently five times using the predefined random seeds {42, 123, 3407, 2023, 5566}. The results reported in the tables are presented as mean values with standard deviations, with the standard deviation ranging from 0.12 percent to 0.37 percent. This ensures the stability and reproducibility of the results.<br><br>This paragraph specifies that all experiments were executed independently five times under identical configurations and describes the use of fixed random seeds to ensure comparability among different runs. The performance values reported in the tables represent the

	average results of five runs, with the corresponding standard deviation range provided to indicate the stable interval of variation in performance. In addition, the experimental configuration, data partitioning, and model initialization were kept consistent across all compared methods, ensuring the fairness of the comparisons.
8. In table 3, Swin-T[2] model comparison is not taken into account. Why?	We sincerely thank the reviewer for the attention to the completeness of model comparisons. In the revised manuscript, additional clarification has been added in Section 4.3 to explain the reason and justification for not including Swin-T in Table 4. The revised text is as follows:<br><br>***Table 4 presents the performance of MViT on the image reconstruction task, where it outperforms other models in both PSNR and SSIM metrics, indicating better modeling capability for spatial structures and edge information. The results validate the effectiveness of the 7×7 partitioning and compensation strategy in maintaining image structural continuity. Since the self-attention mechanism in Swin-T is primarily designed for feature classification and downstream task feature extraction, its official implementation, although publicly available, does not include an image reconstruction or upsampling decoding branch. As a result, it is not possible to directly output pixel-level results for calculating PSNR and SSIM metrics within the same experimental framework.***<br><br>This experiment is designed to evaluate the ability of the method to preserve spatial structure and edge continuity through a pixel level reconstruction task. The original Swin-T architecture was developed for feature level tasks such as classification, detection, and segmentation. The publicly available code does not include a decoding module or an upsampling branch required for pixel level reconstruction, and therefore PSNR and SSIM cannot be calculated under the unified reconstruction framework used in this study.
9. For table 4 and 5, you decide to only report on 2 datasets, not all. Why?	We sincerely thank the reviewer for the attention to the coverage of the experiments. In the revised manuscript, additional explanations have been added in Sections 4.3 and 4.4 to clarify the rationale and representativeness of selecting only two datasets for presentation. The revised text in Section 4.4 is as follows:<br><br>***This section evaluates the independent contributions of each module***

	in structural modeling through two sets of ablation experiments, focusing on the performance contributions of the path construction strategy and the fusion mechanism. Table 2 reports the overall performance of the complete MViT model across multiple datasets for comparison with the baseline networks. Tables 5 and 6 analyze the independent contributions by removing or replacing individual modules under the same training configuration. For clarity and representativeness, these tables only present results for the ImageNet-21k and CIFAR-100 datasets, corresponding to large and medium to small scale classification tasks, respectively. Other datasets exhibit a consistent relative improvement trend under the same experimental settings, with differences controlled within the ±0.3% range, demonstrating stable performance across various data conditions.<br>This addition clarifies the rationale and scientific basis for the table selection. The ImageNet-21k and CIFAR-100 datasets represent large scale and medium scale classification tasks, respectively, and together they reflect model performance under different levels of data complexity and sample size. To avoid redundancy and unnecessary length in the main text, only these two representative results are retained. The remaining datasets follow the same trend under identical configurations, with accuracy differences within 0.3 percent.
10. I think there is a type in table number 1, in the Places 365 row. The sum training set + test set is not equal to total images.	We sincerely thank the reviewer for the careful examination of the table data. After checking again, we confirm that the statistics for the Places365 dataset in the original manuscript contained an error, where the sum of the training set and the validation set did not match the reported total number of images. In the revised manuscript, we have verified and corrected the data according to the official Places365 dataset. The official documentation reports 1,803,460 training images and 36,500 validation images, for a total of approximately 1.839 million images.<br><br>We sincerely apologize for this oversight. In the revised version, we have thoroughly reviewed all dataset statistics presented in Table 1 to ensure that every value is consistent with the official documentation. We again thank the reviewer for this careful and constructive comment, which allowed us to correct this error and improve the accuracy and

	rigor of the dataset information in the paper.
11. It would be interesting to see some more images being divided in different patches as examples.	We sincerely thank the reviewer for the valuable suggestions. These comments have helped us improve both the clarity of the visual presentation and the description of the model structure. In the revised manuscript, additional material has been included in Section 4.8 to provide a clearer illustration of the image patch division and indexing process.<br><br>In particular, the left panel of Figure 9 shows the traversal order of the Moore path on a 16×16 grid, which illustrates how the input image is divided and ordered before it enters the Transformer encoder. Figure 10 presents the positional embedding similarity distribution of the MViT model under a 7×7 patch division, which describes the structural correspondence learned by the model in spatial representation. Through these two forms of visualization, we aim to provide readers with a clearer understanding of the overall mechanism of the method during image partitioning, positional encoding, and spatial alignment.<br><br>We again thank the reviewer for the valuable feedback, which has improved the completeness and readability of the paper.
