# Supplementary material for: MViT: A vision transformer with fractal path reordering and dynamic positional encoding
Source: PLoS One. 2026 Jan 16;21(1):e0340788. doi: 10.1371/journal.pone.0340788 (PMC12810909; doi:10.1371/journal.pone.0340788)
Supplement: Attachment — Submitted filename: Response_to_Reviewers_auresp_2.pdf [file pone.0340788.s002.pdf]

PONE-D-25-38700

MViT: A Vision Transformer with Fractal Path Reordering and Dynamic Positional Encoding

PLOS ONE

To the Academic Editor
Dear Dr. Anil Yaman,

We sincerely thank you and the reviewers for the valuable time and careful evaluation devoted to our manuscript entitled "***MViT: A Vision Transformer with Fractal Path Reordering and Dynamic Positional Encoding***" (Manuscript ID: PONE-D-25-38700).

We have carefully read and fully understood the editorial decision and all comments from the reviewers, and have made corresponding revisions to the manuscript accordingly. With respect to the additional questions raised in the previous round of review, we have addressed them individually in the revised manuscript and provided detailed point-by-point responses in the accompanying "Response to Reviewers" document.

We sincerely appreciate the constructive comments and suggestions provided by the editor and reviewers. These comments have been very helpful in improving the clarity of presentation, logical organization, and overall academic rigor of the manuscript. We believe that the quality of the manuscript has been further improved through this revision.

We hereby submit the revised manuscript for your kind consideration and would be grateful if you and the reviewers could review it again. Should any further revisions be required, we would be pleased to make additional modifications.

Thank you again for your patience and support.

Yours sincerely,
Author Team
School of Design and Art, Shanghai Dianji University
December 18, 2025

Response to Reviewer 3

Dear Reviewer,

Thank you very much for your careful review of our manuscript in this round and for the valuable comments you have provided. We sincerely appreciate your recognition of our previous responses and have given careful consideration to the two additional issues you raised.

Regarding your question about the relationship between the magnitude of performance improvements in Table 2 and the use of the term "significant" , we have re examined the relevant text and revised the wording in the manuscript accordingly. In the revised version, expressions that could potentially lead to a misunderstanding of statistical significance have been explicitly avoided. The performance comparisons are now described in a more accurate manner under identical training settings, ensuring that the statements are consistent with the experimental results.

With respect to your concern about the unclear origin of the "12.6%" value reported in Table 3, we have added explicit clarification in the revised manuscript. This value is now clearly defined as the relative reduction in the accuracy drop of MViT compared to ViT B 16 under rotation perturbations. In addition, the specific calculation based on the experimental results has been provided in the text to avoid potential ambiguity. We have also clarified that this metric is intended to characterize the relative differences in performance degradation under geometric perturbations, rather than to indicate an absolute improvement in classification accuracy.

We sincerely thank you for your careful attention to the details of the manuscript and for your emphasis on precise and rigorous wording. Your comments have been highly valuable in improving the clarity, consistency, and overall academic quality of the paper.

Once again, we greatly appreciate the time and professional effort you have devoted to helping improve this work.

Comment	Response
1.In your paper you mention "MViT demonstrates superior performance across various image classification datasets with different structural complexities, particularly excelling in fine-grained datasets that are sensitive to structure. As shown in Table 2, MViT achieved an accuracy of 84.08% on ImageNet-21k and 90.75% on the Stanford Cars dataset, significantly outperforming both ViT-B/16 and Swin-T." But looking at table 2, I only see a small improvement. Could you clarify how significance was determined?	Thank you very much for the reviewer’s valuable comments on this issue. The concern raised by the reviewer is fully understood and appreciated. Indeed, when considering only the numerical differences reported in Table 2, the performance gains of MViT over ViT-B/16 and Swin-T are relatively modest. In this regard, the original wording "***significantly*** " was not sufficiently precise and could potentially lead to a misunderstanding regarding statistical significance. We appreciate the reviewer’s careful observation. Accordingly, the wording in Section 4.2 has been revised from "***As shown in Table 2, MViT achieved an accuracy of 84.08% on ImageNet-21k and 90.75% on the Stanford Cars dataset, significantly outperforming both ViT-B/16 and Swin-T***" to "***As shown in Table 2, MViT achieves an accuracy of 84.08% on ImageNet-21k and 90.75% on the Stanford Cars dataset, performing better than ViT-B/16 and Swin-T under identical training settings***" in order to avoid any potential ambiguity in the interpretation of the results reported in Table 2.<br><br>It is important to clarify that the original intention of the experimental design and the corresponding description was not to claim statistical significance, but rather to emphasize that the observed performance differences were obtained under strictly consistent experimental conditions and exhibit good reproducibility. As described in Section 4.1, all results are based on fixed data splits and unified training strategies. Multiple independent runs were conducted under fixed random seeds, and the reported results correspond to averaged values, thereby reducing the influence of randomness from individual training runs.<br><br>In addition, it is noted that the notion of stability may have different meanings under different experimental contexts. The results presented in Table 2 are primarily intended to reflect overall performance comparisons on standard classification tasks, with an emphasis on average performance under unified settings. In contrast, stability and robustness under geometric perturbations are more directly evaluated through the rotation disturbance experiments reported in Table 3. Furthermore, the cross-backbone and cross-task evaluations presented in Table 8 further demonstrate that the proposed module maintains consistent performance gains across different architectures and tasks.

	Together, these experiments provide complementary evidence of the stable behavior of the proposed approach under diverse and challenging settings.<br><br>Once again, we sincerely thank the reviewer for highlighting the importance of precise and rigorous wording. This comment has been very helpful in improving the academic clarity and rigor of the manuscript.
2.The sentence above table 3 states: "Compared to ViT-B/16, MViT exhibited a 12.6% reduction in accuracy drop, demonstrating that periodic positional encoding can maintain phase continuity under rotation, alleviating absolute positional index mismatches, and thus improving the stability and generalization ability of the model for rotational and symmetric structures." It was not clear to me how you found 12.6%.	Thank you very much for the reviewer's careful and detailed comment. It is acknowledged that the calculation of this percentage was not sufficiently clarified in the original manuscript, which may indeed have caused confusion for readers. We sincerely appreciate the reviewer's observation.<br><br>The reported value of 12.6% does not refer to an absolute improvement in classification accuracy. Instead, it represents the relative reduction in the accuracy drop of MViT compared to ViT-B/16 under rotation perturbations. This value is directly derived from the results reported in Table 3, as detailed below. In the CIFAR-100 rotation disturbance test, the accuracy of ViT-B/16 decreases from 93.30% to 86.79%, corresponding to a drop of 6.51 percentage points. Under the same testing conditions, the accuracy of MViT decreases from 93.82% to 88.17%, corresponding to a drop of 5.65 percentage points. Accordingly, the relative reduction in the accuracy drop of MViT with respect to ViT-B/16 is calculated as:<br><br>$$\frac{6.51 - 5.65}{6.51} \times 100\% \approx 12.6\%$$<br><br>This metric is intended to quantify the relative difference in performance degradation among different models under rotation perturbations, thereby reflecting their stability under geometric transformations, rather than indicating an absolute gain in classification accuracy.<br><br>Following the reviewer's suggestion, additional clarification has been added to the text above Table 3 in the revised manuscript to explicitly explain the definition and computation of this percentage and to avoid

	potential ambiguity. We again thank the reviewer for the valuable comment, which has helped improve the clarity and rigor of the manuscript.
